# Commentary: Working Memory Load Affects Processing Time in Spoken Word Recognition: Test Retest Reliability of the E-WINDMIL Eyetracking Paradigm

**DOI:** 10.3389/fnins.2021.663930

**Published:** 2021-06-08

**Authors:** Shai Baharav, Gal Nitsan, Boaz M. Ben-David

**Affiliations:** ^1^Baruch Ivcher School of Psychology, Interdisciplinary Center (IDC) Herzliya, Herzliya, Israel; ^2^Department of Communication Sciences and Disorders, University of Haifa, Haifa, Israel; ^3^Department of Speech-Language Pathology, University of Toronto, Toronto, ON, Canada; ^4^Toronto Rehabilitation Institute (TRI), University Health Networks, Toronto, ON, Canada

**Keywords:** visual world paradigm, eye-tracking, working memory load, speech processing and recognition, spoken word identification, cognitive ageing, test retest reliability

A paper by Hadar et al. (2016) published in this journal suggested a new eyetracking paradigm to gauge the cognitive load associated with speech processing. This was accomplished using an adapted version of the eyetracking Visual World Paradigm (VWP). Young normal-hearing listeners completed a spoken word identification task with a concurrent working memory load task as their eye-gaze on the monitor was recorded. While following spoken instructions to touch an object (out of four objects presented on the monitor), the listener was asked to retain either 1 or 4 digits (low load or high load) for later recall. Eye-fixations on a named target-object were compared to fixations on an object whose name had shared phonology (e.g., toweR and toweL), as the spoken word (named target-object) unfolded in time. Results indicated the important role working memory plays in speech perception, even when performed by younger adults in ideal listening conditions.

A recent paper by Nitsan et al. (2019), extended this paradigm and tested the effect of individual differences in working memory capacity on spoken word identification in noise. This adapted paradigm, coined the E-WINDMIL (Eyetracking of Word Identification in Noise Under Memory Increased Load), further highlighted the role of cognitive resources in speech processing, even with younger adults. As researchers increasingly apply the VWP in clinical settings to study speech processing in aging, we were asked whether this paradigm is reliable like the common VWP (Farris-Trimble and McMurray, 2013) across the lifespan. In response, we investigated the test-retest reliability of the E-WINDMIL in both younger and older adults.

Twenty-four younger adults (*M* age = 25.34 years, *SD* = 1.61 years) and 24 older adults (*M* age = 69.04 years, *SD* = 3.61 years) were recruited. Inclusion criteria closely mimicked our previous studies: clinically normal visual acuity, color-vision, pure-tone audiometric thresholds, language proficiency, forward digit span and basic cognitive diagnosis MoCA for older adults. Out of 34 younger adults tested, six were excluded due to loss of eye tracker signal in at least one of the test sessions and four were excluded due to attrition, as they did not return to the second experimental session. The final number of young participants, 24, matched the original Hadar et al. study. Hadar et al.'s methodology (using Hebrew spoken words) was also closely followed, with the following changes: (1) Spoken instructions were mixed with a continuous speech spectrum noise at −4 dB and 0 dB signal-to-noise ratios for young and older adults, respectively; (2) Participants completed the task *twice* following a 2-week interval; and (3) Two image sets and four test versions were created from the original studies to prevent learning of the paradigm stimuli. As such, no participant viewed images nor heard target-object instructions from the first session in the second session (see counterbalancing descriptions here: www.canlab.idc.ac.il/ewindmil).

Growth curve analysis (Mirman, 2014) was used to analyze the time course of fixations on the target-object, from word onset to 200 ms after average word offset, for each age-group separately. Growth Curve Analysis, a multilinear polynomial regression model, is commonly used to model the Visual World Paradigm (Mirman, 2014; Nitsan et al., 2019) demonstrating sufficient statistical power with the sample size chosen for this study. The overall time course of target fixations was captured with a third order (cubic) orthogonal polynomial with fixed effects of load (1 vs. 4 digits preload), and test-session (test vs. retest) on all time terms, and participant random effects on all time terms (note, item order was fully randomized). Onset vs. offset phonemic competitors were modeled separately. Apart from the younger adult offset model, there was no effect of test-session on the time terms in both groups, indicating no significant difference in the rate or number of fixations on the target between the two testing sessions (all *p* > 0.5) in either age group (See Figure 1). However, in the younger adult offset model we witnessed an effect of test-session on the time terms indicating slightly faster fixations to target in the second session (For all tables see Supplementary Materials). This analysis suggests E-WINDMIL's test-retest reliability. The model's syntax and coefficients can be found here: https://github.com/G-Nitsan/GCA-test-retest-2020.

**Figure 1:**
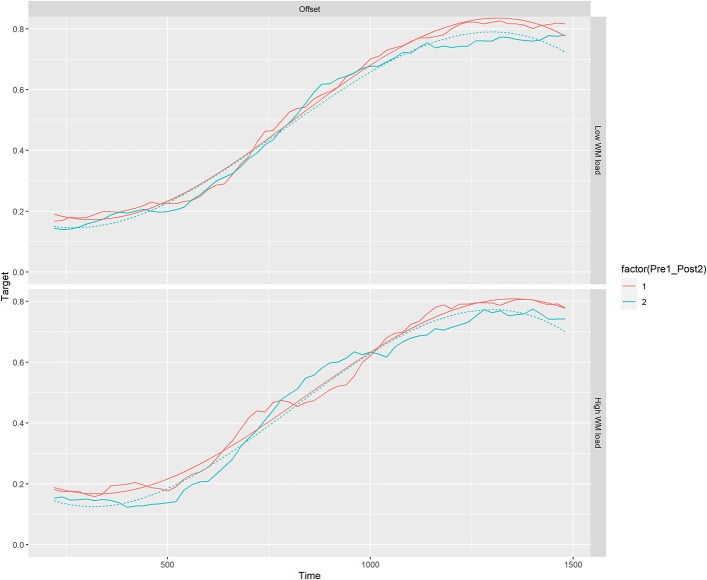
Mean proportion of fixations to the target for older adults in the offset competitor condition compared across test-session 1 and 2 according to low (1 digit) or high (4 digit) working memory preload. The red line indicates test-session 1 and the blue line indicates test-session 2 following a 2 week interval. The model fits (smooth lines) are plotted along with the observed target fixation data.

The test-retest reliability of the E-WINDMIL may facilitate investigating the interaction of cognition and speech processing within clinical settings. Further research is needed to determine how this version of VWP could be used. For example, the E-WINDMILL may serve as a far-transfer gauge of working memory for diagnosis or even cognitive training. This study calls for increased efforts to verify the reliability of tools that may provide new paths for cognitive assessment in aging (Ben-David et al., 2018).

## Author Contributions

SB, GN, and BB-D co-authored the commentary with SB acting as main writer and BB-D as corresponding author. BB-D was head of the lab and architect of the study. GN ran analysis. SB designed and ran the experiment. All authors contributed to the article and approved the submitted version.

## Conflict of Interest

The authors declare that the research was conducted in the absence of any commercial or financial relationships that could be construed as a potential conflict of interest.
