# Supplementary material for: Commentary: Working Memory Load Affects Processing Time in Spoken Word Recognition: Test Retest Reliability of the E-WINDMIL Eyetracking Paradigm
Source: Front Neurosci. 2021 Jun 8;15:663930. doi: 10.3389/fnins.2021.663930 (PMC8224167; doi:10.3389/fnins.2021.663930)

**Younger Adult Onset Competitors**

| *Predictors* | *Estimates* | **Target**  *CI* | *p* |
| --- | --- | --- | --- |
| (Intercept) | -0.22 | -0.55 – 0.12 | 0.209 |
| ot1 | -10.15 | -15.65 – -4.65 | **<0.001** |
| ot2 | -10.08 | -13.94 – -6.22 | **<0.001** |
| ot3 | -4.34 | -5.97 – -2.71 | **<0.001** |
| Pre1_Post2 [2] | -0.19 | -0.44 – 0.06 | 0.142 |
| load [4] | 0.36 | 0.11 – 0.61 | **0.005** |
| Pre1_Post2 [2] * load [4] | -0.11 | -0.46 – 0.25 | 0.557 |
| ot1 * Pre1_Post2 [2] | -2.76 | -6.91 – 1.39 | 0.192 |
| ot1 * load [4] | 5.63 | 1.48 – 9.78 | **0.008** |
| ot2 * Pre1_Post2 [2] | -2.09 | -4.84 – 0.66 | 0.136 |
| ot2 * load [4] | 4.16 | 1.41 – 6.91 | **0.003** |
| ot3 * Pre1_Post2 [2] | -0.93 | -2.19 – 0.33 | 0.148 |
| ot3 * load [4] | 0.89 | -0.37 – 2.14 | 0.167 |
| (ot1 * Pre1_Post2 [2]) * load [4] | -1.54 | -7.41 – 4.33 | 0.606 |
| (ot2 * Pre1_Post2 [2]) * load [4] | -0.82 | -4.71 – 3.07 | 0.679 |
| (ot3 * Pre1_Post2 [2]) * load [4] | -0.28 | -2.05 – 1.50 | 0.761 |

^N^ Subject ^24^

Observations 7104



**Older Adult Onset Competitors**

|  |  |  |
| --- | --- | --- |
|  |  | **Target** |
| *Predictors* | *Estimates* | *CI p* |
| (Intercept) | -0.82 | -1.14 – -0.49 **<0.001** |
| ot1 | -21.27 | -26.36 – -16.17 **<0.001** |
| ot2 | -18.07 | -21.30 – -14.83 **<0.001** |
| ot3 | -6.97 | -8.21 – -5.73 **<0.001** |
| Pre1_Post2 [2] | -0.45 | -0.89 – -0.01 **0.046** |
| load [4] | -0.26 | -0.70 – 0.19 0.257 |
| Pre1_Post2 [2] * load [4] | 1.01 | 0.38 – 1.63 **0.002** |
| ot1 * Pre1_Post2 [2] | -6.44 | -13.48 – 0.60 0.073 |
| ot1 * load [4] | -3.37 | -10.41 – 3.67 0.348 |
| ot2 * Pre1_Post2 [2] | -3.80 | -8.27 – 0.68 0.096 |
| ot2 * load [4] | -2.31 | -6.78 – 2.17 0.312 |
| ot3 * Pre1_Post2 [2] | -1.42 | -3.17 – 0.33 0.111 |
| ot3 * load [4] | -1.38 | -3.13 – 0.36 0.120 |
| (ot1 * Pre1_Post2 [2]) * load [4] | 16.39 | 6.43 – 26.34 **0.001** |
| (ot2 * Pre1_Post2 [2]) * load [4] | 10.67 | 4.34 – 17.00 **0.001** |
| (ot3 * Pre1_Post2 [2]) * load [4] | 4.43 | 1.96 – 6.91 **<0.001** |

^N^ Subject ^24^

Observations 6144

**Younger Adult Offset Competitor**

| *Predictors* | *Estimates* | **Target**  *CI* | *p* |
| --- | --- | --- | --- |
| (Intercept) | -0.26 | -0.45 – -0.08 | **0.006** |
| ot1 | -10.79 | -13.75 – -7.83 | **<0.001** |
| ot2 | -10.02 | -12.00 – -8.04 | **<0.001** |
| ot3 | -4.42 | -5.39 – -3.46 | **<0.001** |
| Pre1_Post2 [2] | 0.35 | 0.10 – 0.59 | **0.006** |
| load [4] | 0.54 | 0.30 – 0.79 | **<0.001** |
| Pre1_Post2 [2] * load [4] | -1.08 | -1.43 – -0.74 | **<0.001** |
| ot1 * Pre1_Post2 [2] | 5.41 | 1.32 – 9.50 | **0.009** |
| ot1 * load [4] | 9.42 | 5.33 – 13.51 | **<0.001** |
| ot2 * Pre1_Post2 [2] | 2.93 | 0.23 – 5.64 | **0.034** |
| ot2 * load [4] | 6.35 | 3.64 – 9.06 | **<0.001** |
| ot3 * Pre1_Post2 [2] | 1.04 | -0.20 – 2.28 | 0.099 |
| ot3 * load [4] | 2.35 | 1.11 – 3.58 | **<0.001** |
| (ot1 * Pre1_Post2 [2]) * load [4] | -17.14 | -22.92 – -11.36 | **<0.001** |
| (ot2 * Pre1_Post2 [2]) * load [4] | -11.25 | -15.08 – -7.42 | **<0.001** |
| (ot3 * Pre1_Post2 [2]) * load [4] | -4.29 | -6.04 – -2.54 | **<0.001** |

^N^ Subject ^24^

Observations 7104



**Older Adult Offset Competitors**

|  |  |  |
| --- | --- | --- |
|  |  | **Target** |
| *Predictors* | *Estimates* | *CI p* |
| (Intercept) | -1.23 | -1.57 – -0.89 **<0.001** |
| ot1 | -27.45 | -32.74 – -22.15 **<0.001** |
| ot2 | -21.92 | -25.23 – -18.60 **<0.001** |
| ot3 | -8.22 | -9.45 – -6.98 **<0.001** |
| Pre1_Post2 [2] | 0.08 | -0.37 – 0.52 0.733 |
| load [4] | 0.28 | -0.16 – 0.72 0.214 |
| Pre1_Post2 [2] * load [4] | -0.51 | -1.14 – 0.12 0.110 |
| ot1 * Pre1_Post2 [2] | 1.85 | -5.20 – 8.90 0.607 |
| ot1 * load [4] | 4.64 | -2.40 – 11.69 0.196 |
| ot2 * Pre1_Post2 [2] | 1.43 | -3.05 – 5.91 0.533 |
| ot2 * load [4] | 3.04 | -1.44 – 7.52 0.184 |
| ot3 * Pre1_Post2 [2] | 0.85 | -0.90 – 2.60 0.339 |
| ot3 * load [4] | 0.86 | -0.89 – 2.61 0.337 |
| (ot1 * Pre1_Post2 [2]) * load [4] | -8.02 | -17.98 – 1.94 0.115 |
| (ot2 * Pre1_Post2 [2]) * load [4] | -5.30 | -11.64 – 1.03 0.101 |
| (ot3 * Pre1_Post2 [2]) * load [4] | -1.95 | -4.43 – 0.52 0.122 |

^N^ Subject ^24^

Observations 6144


Supplement: Supplementary file 1 [file Table_1.docx]
